# Analysis of a Multilevel Diagnosis Decision Support System and Its Implications: A Case Study

**DOI:** 10.1155/2012/367345

**Published:** 2012-12-23

**Authors:** Alejandro Rodríguez-González, Javier Torres-Niño, Miguel A. Mayer, Giner Alor-Hernandez, Mark D. Wilkinson

**Affiliations:** ^1^Centre for Plant Biotechnology and Genomics UPM-INIA, Polytechnic University of Madrid, Parque Científico y Tecnológico de la U.P.M. Campus de Montegancedo, Pozuelo de Alarcón, 28223 Madrid, Spain; ^2^Computer Science Department, Universidad Carlos III de Madrid, Avenida Universidad 30, 28911 Leganés, Spain; ^3^Research Programme on Biomedical Informatics (GRIB), IMIM-Universitat Pompeu Fabra, Dr. Aiguader, 88, 08003 Barcelona, Spain; ^4^Division of Research and Postgraduate Studies, Instituto Tecnológico de Orizaba, Avenida Oriente 9 No. 852 Col. Emiliano Zapata, 94320 Orizaba, ER, Mexico

## Abstract

Medical diagnosis can be performed in an automatic way with the use of computer-based systems or algorithms. Such systems are usually called diagnostic decision support systems (DDSSs) or medical diagnosis systems (MDSs). An evaluation of the performance of a DDSS called ML-DDSS has been performed in this paper. The methodology is based on clinical case resolution performed by physicians which is then used to evaluate the behavior of ML-DDSS. This methodology allows the calculation of values for several well-known metrics such as precision, recall, accuracy, specificity, and Matthews correlation coefficient (MCC). Analysis of the behavior of ML-DDSS reveals interesting results about the behavior of the system and of the physicians who took part in the evaluation process. Global results show how the ML-DDSS system would have significant utility if used in medical practice. The MCC metric reveals an improvement of about 30% in comparison with the experts, and with respect to sensitivity the system returns better results than the experts.

## 1. Introduction

In medical practice, “differential diagnosis” is a systematic approach to determine, based on evidence, the underlying cause of a set of observed symptoms where there are several plausible alternative explanations, and/or to reduce the list of possible diagnoses. 

Medical diagnosis can be performed in an automatic way with the use of computer-based systems or algorithms. Such systems are usually called diagnostic decision support systems (DDSSs) or medical diagnosis systems (MDSs), which fall under the more general category of clinical decision support system (CDSS). The aim of these types of systems is to guide the physicians through the systematic differential diagnosis process. Many CDSS systems are able to provide results even when data is lacking, that is, under uncertainty, and most importantly, they are not limited in the amount of information they can store and are designed to compute results in a few seconds. While previous CDSS systems have often been highly specialized, able to provide differential diagnoses about specific types of diseases, infections, or disorders, this has also been their limitation; they often cannot take into account symptoms or observations outside of those they were designed to examine. However, current computational capacity and the wealth of readily available medical knowledge online make it plausible to create more generalized knowledge bases containing a wider range of medical facts, thus offering the opportunity to create novel CDSS systems that provide diagnoses which take into account a much broader range of observations than traditional systems, and potentially even beyond those of a specialist physician.

It is known that errors occur at several stages of the diagnostic process [1–3]. As such, the implementation of DDSS is an important tool to support physicians dealing with a broad range of diagnostic puzzles. However, if a DDSS is used in real medical practice, and if a physician is expected to rely on it to guide his own diagnoses, it is important to ensure that the accuracy of the system is also sufficiently high. As per Miller et al. [4], a critical area relevant to all medical diagnosis decision systems (MDDSs) is validation, evaluation, and ongoing quality assurance, with accuracy being the most important metric to be evaluated [5, 6]. 

In this paper we present an evaluation of multilevel diagnosis decision support system (ML-DDSS). ML-DDSS was developed with the aim of demonstrating the application of semantic technologies [7] to the creation of medical expert systems. The system is capable of dealing with multilevel diagnoses and provides accurate results based on the evaluation performed here. The system has been designed with the aim of providing diagnoses even in unfavorable conditions, for example, when the number of available observations is limiting, and as such ML-DDSS represents a high-sensitivity medical diagnosis system. This analysis is focused on the results provided by the system, the implication of those results, and a comparison of those results with expert clinical diagnoses of the same cases. A deeper evaluation of the system is conducted using four specific diseases (two common and two less common) which are contained in the knowledge base of ML-DDSS.

The remainder of the paper is organized as follows.  Section 2 outlines relevant literature in the area, focusing on case studies of medical diagnosis systems. In Section 3, ML-DDSS is introduced with a brief explanation about the internal behavior of the system.  Section 4 presents the main results obtained in the evaluation of the system, and these are discussed in comparison with results provided by the physicians involved in the evaluation process. Conclusions and future work are discussed in Section 5.

## 2. State of the Art

Many case studies of medical diagnosis systems have been reported in the literature. The first prominent computer-based clinical consultation system was MYCIN [8] which was designed to function as an aid for infectious disease diagnosis and therapy selection, with an initial emphasis on bacteraemias. Subsequently, other researchers developed medical diagnosis systems such as INTERNIST-I [9], DXplain [10], Isabel [11], Iliad [12], MDX [13], DiagnosisPro [14], and DiagnosMD [15]. These latter medical diagnosis systems explored multiple and complex diagnoses spanning a variety of different medical fields. For example, INTERNIST-I and Iliad are a computer-assisted diagnosis system for internal medicine; DXplain provides access to a medical diagnosis knowledge base via a nationwide computer communications network; Isabel is a recent Web-based clinical decision support system designed to suggest the correct diagnosis in complex medical cases involving adults; MDX is an experimental medical diagnosis system, which currently diagnoses the syndrome called cholestasis; DiagnosisPro is a Web-based medical diagnosis system that provides diagnostic possibilities for 11,000 diseases and 30,000 findings; finally DiagnosisMD attempts medical diagnosis by combining a set of data (symptoms, signs, abnormal test results, Rx data, among others) together with the country, gender, and age of the patient, offering a list of possible diseases using artificial intelligence techniques to refine the diagnosis. In the majority of the aforementioned medical diagnosis systems, different tests were applied in order to measure criterion such as accuracy and sensitivity (to mention only two); however the results suggested that the resulting diagnoses were not sufficiently reliable for clinical applications. The MYCYN therapy recommendations met Stanford experts' standards of acceptable practice only 90.9% of the time, with some variation noted both among individual experts and between Stanford experts and others. INTERNIST-I presented specific deficiencies that needed to be overcome, such as the program's inability to reason anatomically or temporally, explain its reasoning process, and its failure to construct differential diagnoses spanning multiple problem areas. Among the more successful, Isabel suggested correct diagnosis in 48 of 50 cases (96%) with a response time of 2-3 seconds. Moreover, Isabel suggested the correct diagnosis in almost all complex cases involved, particularly with key finding entry. Conversely, tests carried out on Iliad revealed that medical students made fewer diagnostic errors than the automated system and more conclusively confirmed their diagnostic hypotheses when they were tested in their trained domain. However, there is not any information about multilevel diagnosis capabilities of this system.

Outside of accuracy measurements, other studies measure the acceptance, performance, and evaluation of the use of medical diagnosis systems. In [16] the results indicated that physicians are accepting these kinds of applications that enhance their patient management capabilities, but tend to oppose applications in which they perceived an infringement on their management role. In [17] controlled clinical trials assessing the effects of computer-based clinical decision support systems (CDSSs) on physician performance and patient outcomes were systematically reviewed. In [18] a study was carried out to examine how the information provided by a diagnostic decision support system, for clinical cases of varying diagnostic difficulty, affects physicians' diagnostic performance. In the aforementioned studies, the results emphasize two disparate aspects regarding the use of CDSSs: (1) the physicians' diagnostic performance can be strongly influenced by the quality of information and the type of cases involved, and (2) there is a need for human-like interactive capabilities in the CDSS to improve acceptance and use. In [19] a performance evaluation was conducted between four computer-based diagnostic systems. This evaluation was carried out against a set of 105 diagnostically challenging clinical case summaries involving actual patients from ten clinicians. The evaluation results provided a profile of the strengths and limitations of these computer programs. As limitations, the physicians noted the difficulty in identifying the relevant information provided by the systems and filtering the irrelevant information produced. Another study evaluating the performance of diagnosis systems was described in [20] where the authors analyzed the use of Google as a diagnostic aid tool. The results reveal that Google was capable of finding a correct diagnosis in 15 (58%, 95% confidence interval 38% to 77%) cases from the 26 evaluated. In [21], the authors examined how effectively the information provided by a diagnostic system influences the diagnostic performance of a physician. This analysis was performed with a sample of 67 internists using the QMR decision support system to assist them in the diagnosis process of existing clinical cases. The results reveal that diagnostic performance of physicians was significantly higher (*P* < 0.01) on the easy clinical cases and on those cases in which QMR could provide high-quality information.

Over the last years, other works have approached the development of medical differential diagnosis and therapy systems using computational intelligence techniques [22]. These include combined techniques such as neurofuzzy methods [23] that derive fuzzy rules from a set of training data, the application of genetic algorithms (GAs) for rule selection [24], or the unification of genetic algorithms with fuzzy clustering techniques [25]. Nevertheless, few systems address the problem of multilevel diagnosis. Of those that do, the ADONIS approach [26] presents a partial solution to the multilevel diagnosis problem based on the use of description logics, with interesting results but several limitations of the diagnosis process such as not being able to perform normal diagnosis because of the modifications done to perform multilevel diagnosis. The SEDELO approach [27] is similar to ADONIS in that it offers solutions based on description logics, but puts a special emphasis on measurement of the temporal efficiency, as well as addressing the aforementioned limitations of ADONIS.

As can be seen from these related works, there remains a lack of systems and evaluations of systems which provide multilevel diagnosis decision support. In this work we provide an evaluative case study of the application of a recently created multilevel diagnostic system, ML-DDSS, paying special attention to the implications of the evaluation results from both the perspective of potential users (physicians) as well as from the design of the system itself.

## 3. ML-DDSS

The main aim of ML-DDSS is to demonstrate the application of semantic technologies to the creation of expert systems applied to the medical diagnostic process. As described in detail previously [28] ML-DDSS was developed using a knowledge base where the main entities in the diagnostic process included findings (signs and symptoms) and diagnostic tests. The use of such clinical entities is shared with a wide variety of other medical knowledge bases and ontologies [29–31]. However, in addition, the ML-DDSS system takes into account additional clinical features such as the drug regime of the patient in order to calculate which diagnostic alternatives could be discarded due to, for example, drug interactions [32]. While other data elements such as clinical history are certainly relevant to the diagnosis model, these three items—findings (symptoms and signs), tests, and drugs—are considered particularly relevant for performing accurate diagnosis [33]. The internal behavior of the system has been previously explained and discussed [27, 33, 34].

### 3.1. Multilevel

**Figure 1:**
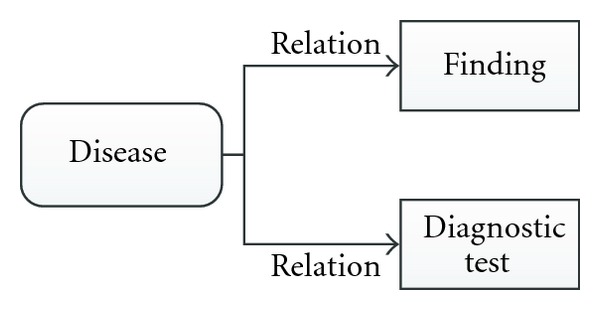
DCM model.

**Figure 2:**
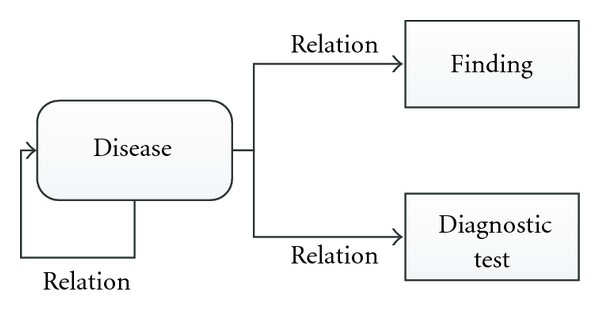
Multilevel DCM model.

The ability to deal with multilevel diagnosis is one of the most interesting aspects of ML-DDSS. The concept of multilevel diagnosis emerges when a disease can be assumed to be a diagnostic element of another disease (acting as a finding). To clarify this, Figures 1 and 2 compare a simple DCM (diagnostic criteria model) with a multilevel DCM.  Figure 2 diagrams the case where the diagnostic criterion of a disease can include combinations of other diseases, along with additional findings and diagnostic results.

**Figure 3:**
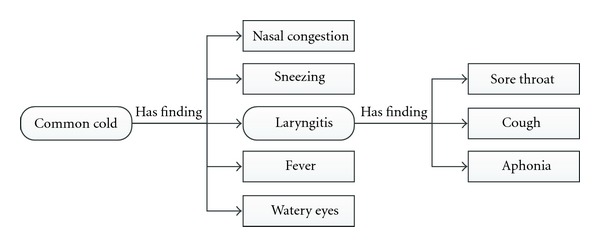
Real representation of multilevel approach.

As a concrete example, Figure 3 shows a reduced (but realistic) multilevel representation of the model of common cold, where laryngitis is a diagnostic indicator of common cold, but in turn has its own diagnostic observations such as sore throat, cough, and aphonia.

In Figure 3, we have a multilevel representation of a disease where the maximum level of depth is *n* = 2, given that we start at level 0 in the main disease (common cold) and we go one level in depth through the laryngitis.

In this paper, we evaluate a system that bases its diagnostic rationale on the assumption that, from a medical perspective, a disease (container) can contain another disease (content) as diagnostic criteria (or contain the findings indicative of that content, which is equivalent). In this case, there is a degree of uncertainty about whether a finding is present or not, and as such, it is necessary to create a mechanism that allows diagnosis of a disease through the findings of another disease. Hence, multilevel diagnosis is one of the main features of this study because it mirrors the true nature of diseases and their associated findings. The ability to create models and systems which can deal with this problem is very important in order to be able to develop accurate diagnosis systems. The current model and system have been designed with this aim as a primary goal.

### 3.2. Sensitivity

High sensitivity refers to the ability of the system to return results even when the number of inputs is at a minimum (*n* > = 1). High sensitivity systems identify all possible solutions matching the input parameters, even when these solutions have a very low probability. These characteristics are advantageous in clinical settings where, in practice, not all data are available to the diagnostic system, and a treatment decision must nevertheless be made; however, such systems have some obvious disadvantages. In particular, with a low number of inputs, and or if those inputs are of low diagnostic specificity (e.g., fever or headache among others), the number of outputs that the system will produce can be very large and therefore of little value in determining the correct diagnosis. This drawback, however, can be addressed using several techniques, the most common being a probabilistic classification of the results [35].

### 3.3. Evaluation Methodology

**Figure 4:**
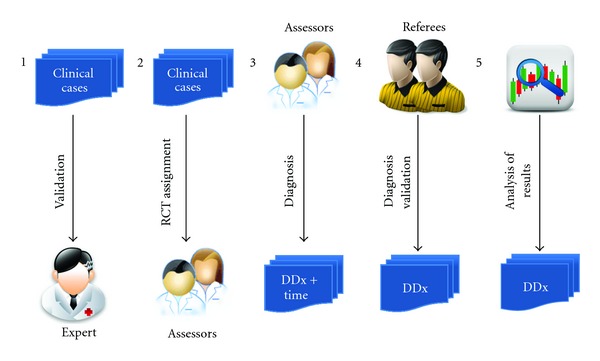
Evaluation process.

The methodology used to evaluate the system is based on the solution of clinical cases, using physicians' diagnosis as a gold standard.  Figure 4 shows a graphical representation of the evaluation process.

In Figure 4, a set of evaluative clinical cases are created by the researchers who developed ML-DDSS. The general format and content of the cases is represented by the following specific case.

A 50-year-old man arrived to the medical center referring symptoms of asthenia, diarrhea, nausea, and vomiting. The patient also suffers vitiligo and hypoglycemia. The patient has lost weight in the last weeks. In the medical center a blood-pressure measurement is done, giving as result a low blood pressure. The diagnostic tests did indicate a decrease in the levels of sodium in the blood, chloride, and bicarbonate and an increase of the potassium. Finally, a hormonal analytics indicates that there is a deficit in aldosterone and in the cortisol basal levels.

Once the cases are created, they are validated by an expert (a physician), who ensures that the cases have been correctly represented. Once the cases have been validated, the researchers assign the clinical cases using a random process (based on randomized controlled trial principles) to a set of assessors (physicians), who provide a differential diagnosis for each clinical case, and the time involved to reach this diagnosis. These results are first verified to ensure that the diagnoses of the diseases provided by the assessors are in the knowledge base of the system. This verification is required because the knowledge base of ML-DDSS contains a sample of only 24 diseases that could be diagnosed. Once the verification process is completed, the diagnoses provided by the assessors and the diagnosis provided by the ML-DDSS system are provided to a set of referees. These referees are then responsible for validating the diagnoses, marking each one as either correct or incorrect. The final step is the analysis and generation of the evaluation results, which is done applying precision, recall (Sensitivity), accuracy, specificity, and MCC (Matthews correlation coefficient [36, 37]). These statistics can be applied over four combinations of data: (1) results validated by referee 1 (R1), (2) results validated by referee 2 (R2), (3) results validated by both referees (intersection R1∩R2), and (4) results validated by one of the referees (union R1 ∪ R2).

The calculation of the different metrics is based on the results provided by the system/assessors and the arbitration process using a confusion matrix as provided in Table 1.

The calculus of these metrics is done using the following formulas:

- Precision = TP/(TP + FP) = A/(A + B).
- Recall = TP/(TP + FN) = A/(A + C).
- Specificity = TN/(TN + FP) = D/(D + B).
- Accuracy = (TP + TN)/(TP + TN + FP + FN) = A/(A + B + C + D).
- MCC = (TP × TN − FP × FN)/((TP + FP) × (TP + FN) × (TN + FP) × (TN + FN)).

## 4. Evaluation Results

Twenty clinical cases were generated for the evaluation. The evaluation process involved 5 assessors and 2 referees. Each clinical case was assigned to three assessors. So, in summary, the parameters of the evaluation were

- clinical cases: 20
- number of assessors: 5
- number of assessors per case: 3
- cases per assessor: 12
- number of referees: 2.

The full results obtained from the evaluation can be found online at [38]. A value of N/A in the tables is interpreted as not applicable. In some circumstances, there are diseases which were neither diagnosed by the assessors nor the ML-DDSS system and therefore have no calculable parameters.

### 4.1. Results of Entire Knowledge Base

**Figure 5:**
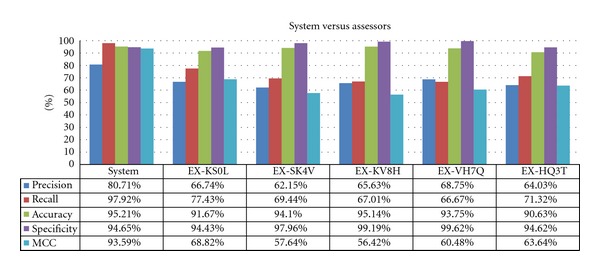
Results of the evaluation (comparison between system and all the assessors).

Figure 5 summarizes the results, showing the values obtained for the entire system, in comparison with the five assessors (anonymized as EX-NNNN in the figure columns). Given that the most constrained calculation for the system is when the values are calculated for the intersection of the arbitration, we have used these as representative values.

When the accuracy is used as a traditional quality metric, the system performs similarly to the best experts. However, the results are quite different from one another, reinforcing the need to use additional metrics in the evaluation. When looking at the MCC, another value that tries to summarize the overall quality, there is a difference of 30% between system and experts. Although the global quality is being measured, the MCC takes into account balance between accuracy and specificity, which is worse in the experts than in the system.

Although the experts were able to provide as many diagnoses as they saw fit, their sensitivity is lower than the automated system. The experts, however, performed better than the system in the specificity metric. However, given that both results are near 95% in the system, it is easier to perform statistically worse than to perform statistically better. This explains the fact that experts have a slight advantage in specificity, while having an important disadvantage in the sensitivity metric. 

These results suggest that the system would be beneficial as a supporting tool for experts, where the system can suggest diagnostics and the experts can confirm them. This would be similar to, for example, a pair of experts where one has the highest sensitivity and the other has the highest specificity; the combination would likely generate better diagnostics than a lone expert. Finally, the precision is much lower for the experts than for the system. Mathematically, this is because the quotient TN (true negative)/TP (true positive) is larger for the experts than for the system, which in practice is because the number of TP is greater for the system (as shown by higher sensitivity values). This has the unexpected consequence that positive predictions from the system are more likely to be true.

To determine if the observed differences are statistically significant (*✓*), Table 2 shows the results of applying a *t*-test to the metrics. The differences between the assessors and the system in precision, accuracy, and specificity were not significant (*✗*).

The conclusions are supported by small confidence intervals for the system, indicating enough data has been gathered to accurately perform the evaluation. It is difficult to extract information about the precision given the wide interval and overlap between experts and system. However, it is possible to extract some conclusions from the MCC and recall metrics, even with wide confidence intervals for the experts, as they do not overlap. More experts or diagnostic cases will be useful in order to narrow those intervals; but the data is useful enough in its current form to draw several conclusions.

### 4.2. Results for Common Diseases

**Figure 6:**
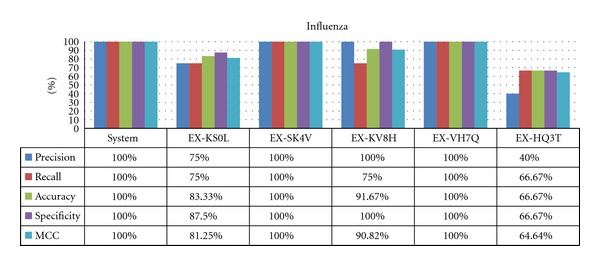
Results for influenza.

**Figure 7:**
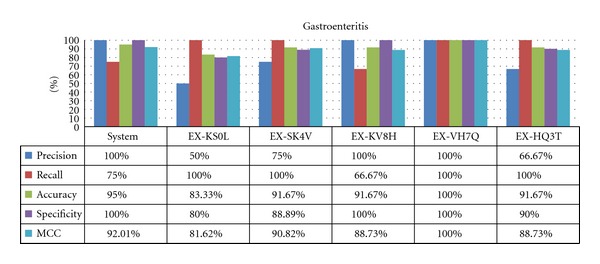
Results for gastroenteritis.

For more frequent diseases, the system can perform as well as experts, in some cases with 100% accuracy as shown for influenza (Figure 6). However, for gastroenteritis (Figure 7), the expert failed to provide the diagnosis in some cases, as sensitivity does not reach 100%. This can probably be attributed to rare cases of the disease, as some experts missed the same cases. The modeling may also be at fault; with only three symptoms in the diagnostic rules for gastroenteritis, it is particularly sensitive to a lack of evidentiary symptoms.

There are also important differences among the assessors, particularly with respect to the MCC and precision metrics, which suggests that the panel of physician evaluators have different levels of familiarity with these diseases. These interevaluator differences were consistent among most of the common diseases, where the best experts in the influenza case match the best in the gastroenteritis case. However, they do not match the global results, which suggest that these experts are worse at diagnosing less common diseases.

The specificity metric is the focal point of the analysis for common diseases; high sensitivity is expected because these diseases are almost always considered during an expert's differential diagnosis. Often, it is more preferable to have a high specificity, in order to rapidly start considering other options in the case where a common disease does not match. Here, the system has surprisingly good results, showing that experts may be biased towards overdiagnosing these common diseases.

### 4.3. Results for Less Common Diseases

**Figure 8:**
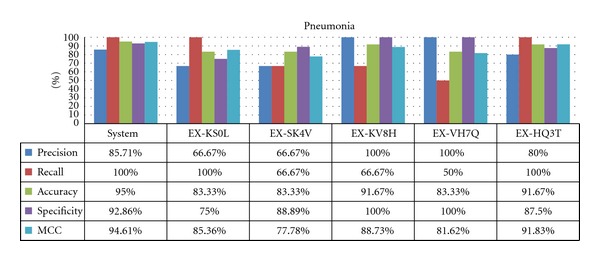
Results for pneumonia.

**Figure 9:**
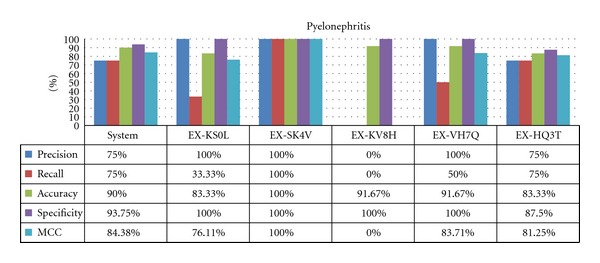
Results for pyelonephritis.

As predicted, there is much less correlation among experts in the rare diseases case, where experts tend to over, or underdiagnose the disease, shown by dramatic differences between sensitivity (recall) and specificity, depending on expert and disease. The results of this behavior are shown for pneumonia (Figure 8) and pyelonephritis (Figure 9). For both diseases, there was an expert whose diagnoses closely matched the system.

It is possible to interpret these results as being indicative of “niche” knowledge, where experts in that niche can accurately diagnose the disease better than other experts. Additionally, the system's overall behavior is very similar to the best expert for each disease, making it comparable to a team of experts covering all disciplines.

Specificity and precision for these rare diseases are generally high, as they usually require more symptoms to be diagnosed, but surprisingly the experts do not rank much higher than the system (which was designed to diagnose a disease with just one matching symptom). The more interesting metric for these diseases is sensitivity, as they can be easily overlooked. In this case, the system shows a clear advantage over the experts.

It is necessary to remark that these results are based on the diseases presented in the examples. Sometimes, rare diseases are characterized for having one or two findings that show the real diagnosis, which means that if you know this specific finding, it becomes easier to accurately diagnose, but if the observation is lacking, then it is more difficult to diagnose. 

## 5. Conclusions and Future Work

The creation of medical diagnosis systems is a problem that has been studied since the early 1960s. Several techniques and technologies have been used in this field, including both knowledge representation tools and algorithms that perform the diagnosis. Most of the approaches are based on the creation of expert systems which capture the knowledge of a set of medical doctors in order to create a clinical decision support system.

While multilevel diagnosis is a well-known approach in the medical field, there are few computational approaches paying special attention to this approach. In this paper we provide an analysis of the results provided by a DDSS system, paying special attention to the metrics that estimate the total accuracy of the system.

The global results show how the DDSS system would have significant utility when used in medical practice. The MCC metric reveals an improvement of about 30% in comparison with the experts. With respect to sensitivity it is also interesting to note that the system returns better results than the experts. As has been stated before, the results confirm that the system would be a good companion for experts, where the system can suggest diagnoses and the experts can confirm them.

A more detailed analysis was conducted using four diseases contained in the knowledge base of ML-DDSS. Influenza and gastroenteritis, selected as common diseases, and pneumonia and pyelonephritis selected to represent less common diseases. Several interesting results were revealed by the analysis of these diseases, including the appearance of “niche” experts from among the assessors. These individual results, therefore, could be used to put together customized diagnostic teams; the analysis of each disease returns very valuable information about how proficient a specific physician is in diagnosing a specific disease. With this information, it would be possible to create cooperative teams based on their individual ability to diagnose a set of diseases.

Future research will be focused on individual analysis of the diseases which make up the knowledge base to improve the quality of the system. Such analyses would provide valuable information that will allow modification of the system's inference rules to improve its accuracy.

## Figures and Tables

**Table 1:** Metrics calculus.

		System/assessor	
		Positive	Negative
Arbitration	Positive	A (TP)	C (FN)
	Negative	B (FP)	D (TN)

**Table 2:** Statistical results.

		Mean	Std. dev.	Confidence interval	*t*-test	Significant differences
Precision	System Assessors mean	0.8071 0.6546	0.29042 0.28082	0.6858–0.9285 0.5372–0.7719	(*t*(46) = −1.850, *P* < 0.05)	*✗*

Recall	System Assessors mean	0.9792 0.7038	0.07058 0.31920	0.9497–1.0000 0.5704–0.8371	(*t*(46) = −4.127, *P* < 0.05)	✓

Accuracy	System Assessors mean	0.9521 0.9306	0.06833 0.06997	0.9235–0.9806 0.9013–0.9598	(*t*(46) = −1.078, *P* < 0.05)	*✗*

Specifity	System Assessors mean	0.9465 0.9716	0.08515 0.03590	0.911–0.9821 0.9566–0.9866	(*t*(46) = 1.331, *P* < 0.05)	*✗*

MCC	System Assessors mean	0.9359 0.6187	0.08975 0.36329	0.8984–0.9734 0.4501–0.7873	(*t*(46) = −4.065, *P* < 0.05)	✓

## References

[1] Kalra J (2004). Medical errors: an introduction to concepts. *Clinical Biochemistry*.

[2] Kalra J (2004). Medical errors: impact on clinical laboratories and other critical areas. *Clinical Biochemistry*.

[3] Berner ES, Graber ML (2008). Overconfidence as a Cause of Diagnostic Error in Medicine. *American Journal of Medicine*.

[4] Miller RA, Pople HE, Myers JD (1982). Internist-I, an experimental computer-based diagnostic consultant for general internal medicine. *The New England Journal of Medicine*.

[5] Swets JA (1988). Measuring the accuracy of diagnostic systems. *Science*.

[6] Huguet J, Castineiras MJ, Fuentes-Arderiu X (1993). Diagnostic accuracy evaluation using ROC curve analysis. *Scandinavian Journal of Clinical and Laboratory Investigation*.

[7] Berners-Lee T, Hendler J, Lassila O (2001). The semantic web. *Scientific American*.

[8] Yua VL, Buchananb BG, Shortliffe EH (1979). Evaluating the performance of a computer-based consultant. *Computer Programs in Biomedicine*.

[9] Miller RA, Pople HE, Myers JD (1982). Internist-I, an experimental computer-based diagnostic consultant for general internal medicine. *The New England Journal of Medicine*.

[10] Barnett GO, Cimino JJ, Hupp JA, Hoffer EP (1987). DXplain: experience with knowledge acquisition and program evaluation. *Proceedings of the Annual Symposium on Computer Application in Medical Care*.

[11] Graber ML, Mathew A (2008). Performance of a web-based clinical diagnosis support system for internists. *Journal of General Internal Medicine*.

[12] Lincoln MJ, Turner CW, Haug PJ (1991). Iliad training enhances medical students’ diagnostic skills. *Journal of Medical Systems*.

[13] Mittal S, Chandrasekaran B, Smith J (1979). Overview of MDX-A system for medical diagnosis. *Proceedings of the Annual Symposium on Computer Application in Medical Care*.

[14] Aronson A (1997). DiagnosisPro: the ultimate differential diagnosis assistant. *Journal of the American Medical Association*.

[15] DiagnosMD Software DiagnosMD. http://www.diagnosmd.com/index.php.

[16] Teach RL, Shortliffe EH (1981). An analysis of physician attitudes regarding computer-based clinical consultation systems. *Computers and Biomedical Research*.

[17] Hunt DL, Haynes RB, Hanna SE, Smith K (1998). Effects of computer-based clinical decision support systems on physician performance and patient outcomes: a systematic review. *Journal of the American Medical Association*.

[18] Berner ES, Maisiak RS, Cobbs CG, Taunton OD (1999). Effects of a decision support system on physicians’ diagnostic performance. *Journal of the American Medical Informatics Association*.

[19] Berner ES, Webster GD, Shugerman AA (1994). Performance of four computer-based diagnostic systems. *The New England Journal of Medicine*.

[20] Tang H, Ng JHK (2006). Googling for a diagnosis—use of Google as a diagnostic aid: internet based study. *British Medical Journal*.

[21] Berner ES, Maisiak RS, Cobbs CG, Taunton OD (1999). Effects of a decision support system on physicians’ diagnostic performance. *Journal of the American Medical Informatics Association*.

[22] Zhao W, Yanxiang H, Hui J A model of intelligent distributed medical diagnosis and therapy system based on mobile agent and ontology.

[23] Nauck D, Kruse R (1997). A neuro-fuzzy method to learn fuzzy classification rules from data. *Fuzzy Sets and Systems*.

[24] Ishibuchi H, Nakashima T, Murata T (1999). Performance evaluation of fuzzy classifier systems for multidimensional pattern classification problems. *IEEE Transactions on Systems, Man, and Cybernetics B*.

[25] Setnes M, Babuska R (1999). Fuzzy relational classifier trained by fuzzy clustering. *IEEE Transactions on Systems, Man, and Cybernetics B*.

[26] Rodríguez-González A, García-Crespo Á, Colomo-Palacios R, Gayo JEL, Gómez-Berbís JM, Alor-Hernández G (2011). Automated diagnosis through ontologies and logical descriptions: the ADONIS approach. *International Journal of Decision Support System Technology*.

[27] Rodríguez-González A, Labra-Gayo JE, Colomo-Palacios R, Mayer MA, Gómez-Berbís JM, García-Crespo A (2012). SeDeLo: using semantics and description logics to support aided clinical diagnosis. *Journal of Medical Systems*.

[28] Rodríguez-González A, Hernandez-Chan G, Colomo-Palacios R (2012). Towards an ontology to support semantics enabled diagnostic decision support systems. *Current Bioinformatics*.

[29] Bertaud-Gounot V, Duvauferrier R, Burgun A (2012). Ontology and medical diagnosis. *Informatics for Health and Social Care*.

[30] Burgun A, Bodenreider O, Jacquelinet C (2005). Issues in the classification of disease instances with ontologies. *Studies in Health Technology and Informatics*.

[31] Peelen L, Klein MCA, Schlobach S, De Keizer NF, Peek N Analyzing differences in operational disease definitions using ontological modeling.

[32] Rodriguez-Gonzalez A, García- Crespo A, Colomo-Palacios R, Gomez- Berbis JM, Jiménez-Domingo E (2011). Using ontologies in drug prescription: the semMed approach. *International Journal of Information and Decision Sciences*.

[33] García-Crespo Á, Rodríguez A, Mencke M, Gómez-Berbís JM, Colomo-Palacios R (2010). ODDIN: ontology-driven differential diagnosis based on logical inference and probabilistic refinements. *Expert Systems with Applications*.

[34] Rodríguez-González A, García-Crespo Á, Colomo-Palacios R, Gayo JEL, Gómez-Berbís JM, Alor-Hernández G (2011). Automated diagnosis through ontologies and logical descriptions: the ADONIS approach. *International Journal of Decision Support System Technology*.

[35] Sox HC (1986). Probability theory in the use of diagnostic tests: an introduction to critical study of the literature. *Annals of Internal Medicine*.

[36] Matthews BW (1975). Comparison of the predicted and observed secondary structure of T4 phage lysozyme. *Biochimica et Biophysica Acta*.

[37] Baldi P, Brunak S, Chauvin Y, Andersen CAF, Nielsen H (2000). Assessing the accuracy of prediction algorithms for classification: an overview. *Bioinformatics*.

[38] Rodríguez-González A Full results of PhD thesis evaluation. http://nadir.uc3m.es/alejandro/pubs/evmds/fullresults.pdf.

